# Effectiveness and safety of different electromagnetic stimulation therapies in treating post-stroke insomnia: A network meta-analysis of randomized controlled trials

**DOI:** 10.1371/journal.pone.0327544

**Published:** 2025-07-03

**Authors:** Shuan-Zhu Sun, Fan Yuan, Lie-Xi Song, Xiao-Zhong Liu, Tao Zhong, De-Liang Zhu, Ke-Yu Chen, Wei-Cheng Wang, Ruo-Yang Li

**Affiliations:** 1 Geriatric Diseases Institute of Chengdu, Department of Rehabilitation, Chengdu Fifth People’s Hospital (The Second Clinical Medical College, Affiliated Fifth People’s Hospital of Chengdu University of Traditional Chinese Medicine), Chengdu, Sichuan, China; 2 Department of Dermatology, Hospital of Chengdu University of Traditional Chinese Medicine, Chengdu, Sichuan, China; 3 Department of traditional Chinese medicine, Chengdu Second People’s Hospital, Chengdu, Sichuan, China; Federal University of Alfenas, BRAZIL

## Abstract

**Objectives:**

To evaluate the efficacy and safety of different electromagnetic therapies for the treatment of post-stroke insomnia (PSI). Thus, we conducted a network meta-analysis to provide evidence-based insights for clinical practice.

**Methods:**

Databases such as PubMed, Excerpt Medica Database (Embase), Cochrane Library Central Register of Controlled Trials, APA PsycInfo, China National Knowledge Infrastructure Database, Wanfang, and SinoMed were used to retrieve randomized controlled trials (RCTs) on electromagnetic therapy for PSI, with a search deadline of Sep 2024 for each database. The Cochrane bias risk assessment tool was used to evaluate the quality of the included RCTs. Stata was used for network meta-analysis.

**Results:**

We included 28 RCTs involving 2353 patients across 12 different treatment regimens. The surface under the cumulative ranking results showed that the ranking of Pittsburgh sleep quality index decline was: cranial electrotherapy stimulation>low frequency repetitive transcranial magnetic stimulation (LF-rTMS)>infra-low frequency repetitive transcranial magnetic stimulation (ILF-rTMS)>fastigial nucleus stimulation (FNS)>transcranial direct current stimulation (tDCS)>low frequency electric stimulation (L-FES)>high frequency repetitive transcranial magnetic stimulation (HF-rTMS)>middle frequency repetitive transcranial magnetic stimulation (MF-rTMS)>sham stimulation (SS)>common treatment (CT); Ranking of Hamilton depression scale decline degree: HF-rTMS > LF-rTMS > tDCS > SS>continuous theta-burst stimulation (cTBS)>MF-rTMS > CT; national Institute of health stroke scale decline ranking: HF-rTMS > LF-rTMS > SS > L-FES>electroencephalographic biomimetic stimulation>CT > cTBS; Clinical total effective rate ranking: LF-rTMS > FNS > ILF-rTMS > L-FES > CT>repetitive transcranial acupuncture stimulation.

**Conclusions:**

Different electromagnetic therapies can effectively improve sleep quality in PSI patients, and the efficacy and safety of LF-rTMS are significant. However, owing to the limitations of this study, the efficacy ranking cannot fully explain the advantages and disadvantages of clinical efficacy. In the future, additional multicentre, large-sample, double-blind, clinical, and randomized controlled trials are required to supplement and demonstrate the results of this study.

**Strengths and limitations of this study:**

This is the first study to conduct network meta-analysis on PSI treatment with different electromagnetic therapies. Simultaneously, we refined the classification based on different frequency patterns of the same therapy, and the results can serve as a reference for clinical workers. This study had some limitations: A large proportion of low-quality literature may lead to biased results; Lack of subgroup analysis, mainly because the number of studies included was not very high, and the quality of most studies was low. Basic information such as stroke site and onset time were not detailed, which may increase the possibility of inconsistency and clinical heterogeneity. After all, detailed subgroup analysis based on the stage or location of stroke can provide more meaningful clinical guidance.

## Introduction

Stroke is a common medical condition worldwide and is characterised by high incidence, recurrence, disability, and mortality rates. Motor dysfunction, cognitive dysfunction, swallowing dysfunction, central nervous system pain, sleep disorders, and other sequelae caused by its onset seriously affect the lives of patients and their families. Post-stroke insomnia (PSI) is a common sequela in stroke patients, and more than half of acute stroke patients develop sleep disorders including insomnia [1]. PSI is associated with environmental factors, psychological factors, and the stroke site. A stroke in the pons leads to almost complete sleep loss, whereas a stroke in the thalamus results in the disruption of brain waves, leading to insomnia. Stroke in the supratentorial, left hemisphere, or paramedian colliculus brain positions can reduce non-rapid eye movements, whereas stroke in the right hemisphere can reduce rapid eye movements [2,3]. Long-term low-quality sleep increases the risk of relapse and incidence of post-stroke depression [4].

In clinical practice, stroke accompanied by sleep disorders is more common and may affect more than 50% of patients. Specific clinical symptoms can occur in stroke patients in specific areas. Patients are more likely to experience sleep disorders if a stroke affects the thalamus, hypothalamus, basal frontal lobe, and orbital cortex in the basal ganglia. The sleep cycle and circadian rhythm are related to the hypothalamic supraoptic nucleus, and a reduced sleep cycle in stroke patients may be related to the medial pontoparietal cortex damage [5]. Among stroke patients, the incidence of insomnia is higher in women than in men [6]. Living alone and being older may also increase the risk of developing PSI [7]. Patients with right hemisphere strokes self-reported experiencing more insomnia than those with left hemisphere strokes [8]. Currently, drug therapy is the first-line treatment for PSI. Traditional drugs for treating insomnia can be divided into sedative (phenobarbital) and non-sedative hypnotic (diazepam, fluazepam, and estazolam) drugs. At present, estazolam, diazepam, and dexzopiclone are commonly used to treat insomnia in clinical practice. Non-sedative hypnotic drugs mainly include antidepressants, such as 5-HT, and norepinephrine reuptake inhibitors, antipsychotics, cytokines, and new hypnotic drugs, which are being used to treat insomnia. Besides drug therapy, patients are increasingly considering emerging physical electromagnetic treatments. Common therapies with different mechanisms of actions, such as repetitive transcranial magnetic stimulation (rTMS), transcranial direct current stimulation (tDCS), and low-frequency electric stimulation (L-FES), which have excellent efficacy and fewer adverse reactions, have gradually gained the favour of clinical workers. rTMS, the most used noninvasive neural regulation technology, utilizes constantly changing magnetic fields to generate induced electric fields in the cerebral cortex, thus regulating a series of physiological neuronal activities [9]. Unlike rTMS, tDCS increases or decreases neuronal excitability by inputting a weak and constant direct current into the brain, leading to changes in brain function [10]. Currently, numerous clinical studies have confirmed the effectiveness of electromagnetic therapy for PSI treatment, but there are only a few relevant network meta-analyses that have systematically summarized its clinical efficacy. Network meta-analysis, as an emerging statistical method, can comprehensively compare multiple treatment options and provide more reliable decision-making basis for researchers and clinical doctors. By considering both direct and indirect comparisons, network meta-analysis can fully utilize existing research data to provide scientific support for patients in selecting the best treatment plan.

We conducted a network meta-analysis to evaluate the effectiveness and safety of different electromagnetic therapy interventions for PSI using Pittsburgh sleep quality index (PSQI), Hamilton depression scale (HAMD), national Institute of health stroke scale (NIHSS), clinical total effective rate, and adverse reactions as outcome indicators. The research results are expected to provide valuable insights for clinical workers.

## Method

### Registration

This meta-analysis is registered on the international prospective register of systematic reviews (registration number CRD42023465844).

### Retrieval Strategy

Our search strategy combined thematic keywords and free text terms, with adjustments made based on the search results. The search period was from establishment of the database to Sep 2024. The following databases were used to retrieve data: PubMed, Excerpt Medica Database (Embase), Cochrane Library Central Register of Controlled Trials, APA PsycInfo, China national knowledge infrastructure database, Wanfang, and SinoMed. The search terms used were: Disorders of Initiating, Maintaining Sleep, Early Awakening, Awakening, Early, Insomnia, Sleep Initiation Dysfunction, Sleeplessness, Insomnias, Stroke, Strokes, Cerebrovascular Accident, Cerebrovascular Accidents, Cerebrovascular Apoplexy, Cerebrovascular, et al. The specific search formula is provided in the Supplement Appendix.

### Literature inclusion criteria

1. The research type was a randomized controlled trial (RCT) regardless of whether allocation concealment or blinding methods were used. 2. The study subjects were diagnosed with stroke through imaging examinations such as brain CT and MRI. 3. Stroke patients with insomnia at baseline were included. 4. Treatment plan: The control group received conventional therapy (conventional antidepressant and insomnia medication therapy) or sham stimulation therapy (SS), while the treatment group received electromagnetic therapy. 5. Outcome measures: The main measures were PSQI, HAMD, and NIHSS scores. Secondary indicator: Clinical total effective rate (total effective rate = [(cured + significantly effective + effective) cases ÷ total cases] × 100%). The clinical total effective rate refers to the efficacy evaluation criteria for insomnia formulated in the Guiding Principles for Clinical Research of New Chinese Medicines [11]. Cured: The sleep time returns to normal or the nighttime sleep time is more than 6 hours, the sleep is deep and energetic after waking up; Significantly effective: the total sleep duration increased by more than 3 hours compared to before, and there was no discomfort; Effective: The total sleep hours have increased compared to before, but the increase is less than 3 hours; Invalid: The total sleep hours have not improved compared to before.

### Exclusion criteria for literature

1. The research types were overview, cohort studies, animal experiments, case studies, basic research, cross-sectional studies, and case reports. 2. The research subjects are not humans or patients who have not been diagnosed with stroke. 3. Stroke patients who did not suffer from insomnia at baseline were included. 4. Treatment plan: The treatment group did not use electromagnetic therapy. 5. Literature with outcome indicators that did not meet the inclusion criteria. 6. Others: Unavailable full-text literature; literature where information could not be extracted, unavailable complete original data, and unsuccessful data requests.; duplicate publications.

### Literature screening and data extraction

Two trained researchers screened the relevant literature separately. The initial screening was based mainly on titles and abstracts. After the initial screening, we searched for literature that met the inclusion criteria and read the full text. If necessary, the original author was contacted to avoid data omission. If the evaluation results in the literature were inconsistent, a third party was invited to participate in the discussion to resolve any issues. Endnote and Excel software were used for literature management, and data extraction included basic characteristics, intervention measures, treatment courses, and outcome indicators of the included literature cases. All continuous data were included in the calculation of changes between before and after treatment values, which involved determining the difference in indicators after treatment compared to before treatment. If these calculations were not presented in the original text, they were independently computed. The formula is as follows [12], corr is usually 0.5.


$${SD}_{E\;change}=\sqrt{{SD}_{E\;baseline}^{2}+{SD}_{E\;final}^{2}-(2\times Corr\times {SD}_{E\;baseline}\times {SD}_{E\;final})}$$

$${Mean}_{E\;change}={Mean}_{E\;final}-{Mean}_{\begin{array}{c} E\;baseline \end{array}}$$

### Risk assessment

The included studies were evaluated according to the bias risk assessment tool recommended in the Cochrane Handbook 5.1.0, which includes seven aspects: random sequence generation in the literature; allocation concealment; implementation of blinding; whether blinding was implemented for outcome evaluation; completeness of outcome data; whether the results were selectively reported; and whether there were other biases. RevMan5.3 software was used to draw a literature quality evaluation chart.

### Statistical analysis

A network meta-analysis was conducted using STATA 14.0 software (Stata Corporation, Lakeway, Texas, USA), with continuity indicators (PSQI, NIHSS, HAMD) using mean difference (MD) as the effect measure. For the binary variable indicator (clinical total effective rate), the relative risk (RR) was used as the effect measure, and the corresponding 95% confidence interval (CI) was calculated. STATA was also used to draw the network evidence relationship maps, forest maps, hierarchical probability maps, funnel maps, and corresponding statistics. A lack of statistically significant differences (P > 0.05) indicated an absence of global inconsistency. This study used surface under the cumulative ranking (SUCRA) to calculate the cumulative ranking probability of each treatment plan. The larger the SUCRA value, the larger the area under the curve of the cumulative probability ranking chart, which indicated better effectiveness of the intervention measure.

## Results

### Literature search results

**Fig1:**
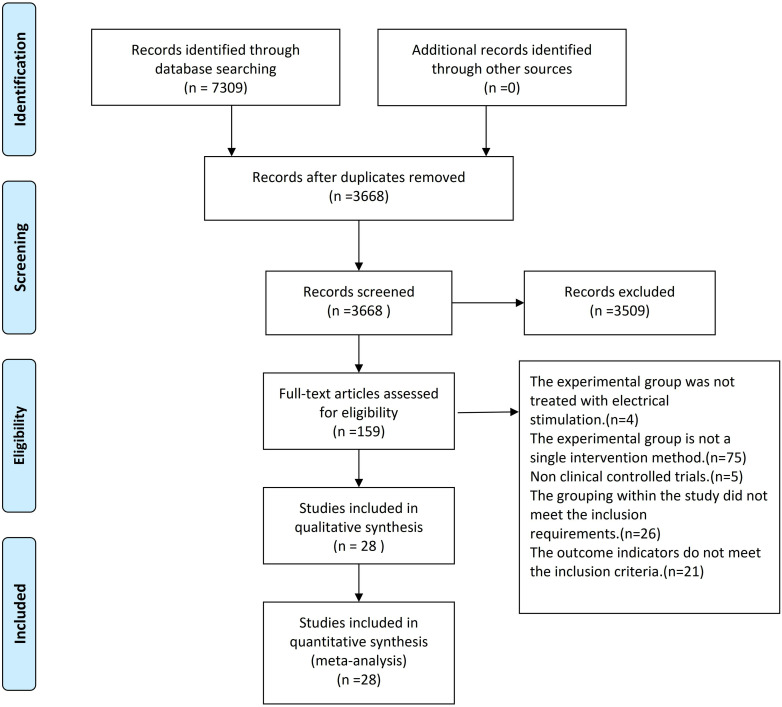
Literature search process.

A total of 7309 articles were obtained from the literature search. After initial screening, 3641 articles were excluded, leaving 3668 articles. After reading the titles and abstracts, 3509 articles were excluded, and after reading the entire text, 131 articles were excluded. The total number of articles included in this study was 28 [13–40]. Twelve therapies were used: common treatment (CT), L-FES, infra-low-frequency repetitive transcranial magnetic stimulation (ILF-rTMS), low-frequency repetitive transcranial magnetic stimulation (LF-rTMS), fastigial nucleus stimulation (FNS), continuous theta-burst stimulation (cTBS), sham stimulation (SS), middle-frequency repetitive transcranial magnetic stimulation (MF-rTMS), high-frequency repetitive transcranial magnetic stimulation (HF-rTMS), cranial electrotherapy stimulation (CES), tDCS, and continuous theta burst stimulation (cTBS). The literature search process and results are shown in Fig 1.

### General characteristics of literature

The results of the 28 included studies [13–40] were published between 2010 and 2023, with a total of 2353 patients. The treatment groups included in the studies were all treated with physical intervention therapy primarily based on electromagnetic therapy, with nine studies using clinical efficacy as the outcome measure [20,22,23,25,26,29,31,37,38], 18 studies using the PSQI score as the outcome measure [13–19,21,22,27,28,30–33,35,39,40], seven studies using HAMD as the outcome measure [13,17–19,24,34,36], and seven studies using the NIHSS as the outcome measure [16,22,24,30,33,35,36]. Basic information on the included studies is presented in Table 1.

**Table 1:** Basic characteristics of the included trials.

Author	Year	Number of patients		Age (years)		Male/female	Treatment		Intervention period	Outcome indicator
		I	C	I	C		I	C		
Chen LP [13]	2020	32	31	64.06 ± 6.82	65.16 ± 9.18	44/19	HF-rTMS+Escitalopram	Escitalopram+SS	4W	PSQI, HAMD
Chen WY [14]	2022	69	64	63.3 ± 9.6	62.6 ± 11.8	91/42	LF-rTMS	Routine rehaliltation	4W	PSQI
Ding L [15]	2020	46	46	70 ± 4	72 ± 4	58/34	LF-rTMS+Bailemian capsule	SS+Bailemian capsule	2W	PSQI, Adverse reactions
Dong Y [16]	2022	60	60	64.85 ± 11.96	63.67 ± 11.39	87/33	L-FES	Routine rehaliltation	12W	PSQI, NIHSS
Gu B [17]	2022	20(The number after shedding 2 cases)	20(The number after shedding 2 cases)	54.2 ± 12.66	58.6 ± 12.58	28/12	tDCS	SS	4W	PSQI, HAMD
Han Q [18]	2016	50	50	56.8 ± 7.3	56.9 ± 7.2	56/43	HF-rTMS	Routine rehaliltation	12W	PSQI, HAMD
He YG [19]	2015	30	30	56.45 ± 9.62	58.52 ± 10.62	40/20	MF-rTMS+Escitalopram	Escitalopram	8W	PSQI, HAMD
Hou ZT [20]	2018	30	30	61 ± 5	62 ± 5	29/31	rTAS	Diazepam	4W	Clinical total effective rate
Huang DX [21]	2022	45	45	61.06 ± 4.65	61.2 ± 4.69	43/47	LF-rTMS	Eszopiclone	2W	PSQI
Huang L [22]	2019	31	31	61.14 ± 10.28	60.48 ± 9.45	30/32	FNS	Routine rehaliltation	5W	PSQI, NIHSS, Clinical total effective rate
Huang SF [23]	2014	34	34	58.75 ± 4.17	58.96 ± 4.32	39/29	L-FES	Routine rehaliltation	2W	Clinical total effective rate
Li GH [24]	2023	30	30	52.27 ± 5.39	53 ± 5.38	23/37	cTBS+Zolpidem	Zolpidem	2W	HAMD, NIHSS
Li J [25]	2021	70	70	56.33 ± 4.1	56.14 ± 3.94	71/69	ILF-rTMS	Routine rehaliltation	3M	Clinical total effective rate, Adverse reactions
Li X [26]	2023	45	45	69.91 ± 7.26	69.96 ± 7.31	51/39	LF-rTMS+Alprazolam	Alprazolam	2W	Clinical total effective rate
Li Y [27]	2015	30	30	–	–	–	CES+Estazolam	SS+Estazolam	4W	PSQI
Liu SW [28]	2017	23	23 23	62.43 ± 10.06	63.35 ± 11.7 63.78 ± 11.29	44/25	Routine rehaliltation	SS FNS	4W	PSQI
Liu WP [29]	2020	60	60	56.33 ± 4.1	56.14 ± 3.94	61/59	FNS+Estazolam+Donepezil	Estazolam+Donepezil	4W	Clinical total effective rate
Qi WY [30]	2022	46	45	63.12 ± 6.07	63.75 ± 5.92	53/38	LF-rTMS	Alprazolam	4W	PSQI, NIHSS, Adverse reactions
Tang L [31]	2014	40	40	63 ± 11	65 ± 10	43/37	L-FES	Estazolam	4W	PSQI, Clinical total effective rate
Tian Y [32]	2021	28	29	65.97 ± 10.51	65.9 ± 9.5	29/28	HF-rTMS	SS	2W	PSQI
Wang CL [33]	2020	30	30	63.5 ± 7.5	64.4 ± 5.6	34/26	L-FES	Routine rehaliltation	1M	PSQI, NIHSS
Wang YN [34]	2017	60	60	72.61 ± 10.23	72.48 ± 9.63	76/44	LF-rTMS	Fluoxetine	4W	HAMD
Xu D [35]	2021	30	28	65.7 ± 6.1	64.2 ± 5.9	35/23	LF-rTMS	Alprazolam	4W	PSQI, NIHSS, Adverse reactions
Yan TT [36]	2010	20 20	20 20	68.65 ± 7.62 69.65 ± 5.81	68.7 ± 8.94 67.25 ± 9.15		HF-rTMS+FMT LF-rTMS+FMT	SS+FMT CT+FMT	7D	HAMD, NIHSS
Yang XJ [37]	2016	70	70	58.34 ± 6.22	58.24 ± 4.82	69/71	L-FES	Alprazolam	4W	Clinical total effective rate
You F [38]	2017	40	40	63 ± 11	65 ± 10	43/37	L-FES	Estazolam	4W	Clinical total effective rate
Zhong ZG [39]	2022	50	50	63.86 ± 8.785	62.88 ± 7.991	61/39	LF-rTMS	Routine rehaliltation	7D	PSQI
Zhu MY [40]	2019	30	30	65.97 ± 10.51	65.9 ± 9.5	29/31	ILF-rTMS	SS	10D	PSQI

### Risk assessment

**Fig 2:**
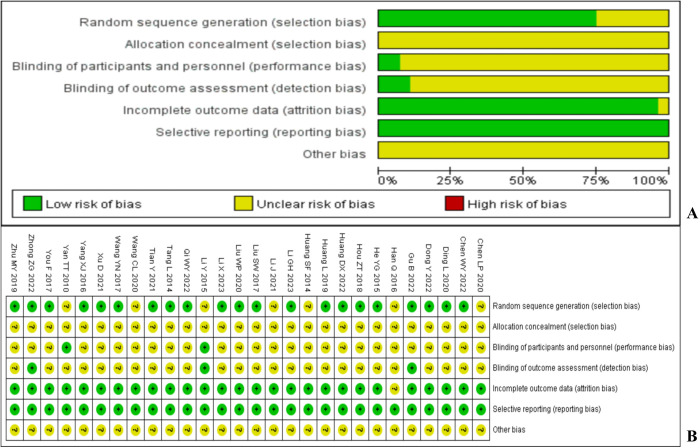
Risk of bias ( A: Risk of bias graph; B: Risk of bias summary).

Among the 28 studies included, 21 studies [14–17,19–22,24,26,28–32,34,35,37–40] generated random sequences using low-risk allocation methods such as random number table and random envelope method. The remaining studies did not specify specific random methods and were classified as unclear risks; All studies have not indicated hidden allocation and are considered unclear risks; Two studies [27,36] mentioned the use of double blinding in terms of performance bias, where both the subjects and the executors were unaware of the grouping situation, both were low-risk, and the remaining studies were unclear risk; Three studies [17,27,39] mentioned that the evaluators were unaware of the grouping situation in terms of assessing bias, both of which were low-risk, while the remaining studies were all unclear risk; Due to the inability to obtain registration plans for 28 studies, the risk of reporting bias was determined by reviewing the methodology and results sections of the included studies. 27 studies were fully reported, while 1 study’s [18] total number of people in the control group is not consistent with the total number of men and women, making it impossible to determine whether a complete report have be made. In terms of selective reporting of research results, selective reporting is not explicitly mentioned in the text, and the included studies are all low-risk. It is unclear whether there are other bias risks in all studies. The bias risk assessment chart for the included studies is shown in Fig 2.

## PSQI

### Evidence Network

**Fig 3:**
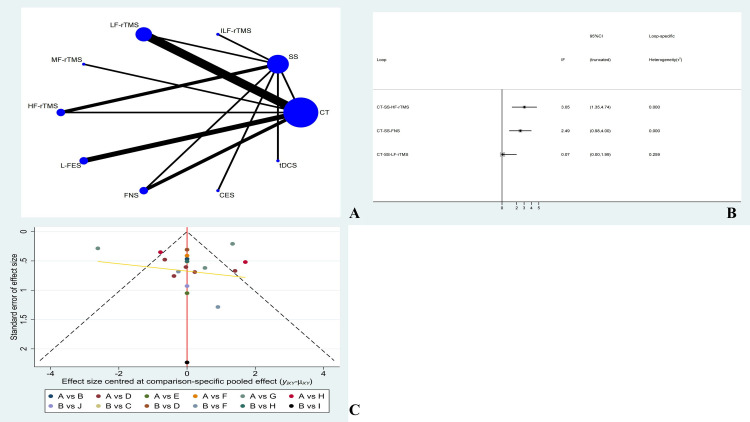
PSQI ( A: Network diagram of PSQI; B: PSQI inconsistency testing; C: Publication bias graph of PSQI).

Eighteen studies reported PSQI involving ten treatment options. The size of the dots represents the sample size used for the intervention measure, and the thickness of the lines represents the number of RCTs using two treatment plans with three closed loops formed. The number of studies comparing CT with LF-rTMS treatment was the highest. The evidence network is shown in Fig 3A.

### Inconsistency

The 10 treatment options for PSQI form three triangular loops. The overall inconsistency test results showed P = 0.2486 > 0.05, indicating no overall inconsistency. The sources of inconsistency were as follows: 3.05 (CT-SS-HF-rTMS) with a 95% CI of (1.35, 4.74); 2.49 (CT-SS-FNS), 95% CI of (0.98,4.00); 0.07 (CT-SS-LF-rTMS), 95% CI of (0.00, 1.99). Generally, when the 95% CI of the inconsistency factor reaches zero, it indicates the absence of statistical inconsistency. In this study, the 95% CI of the two closed-loop systems did not reach zero. We used the node splitting method combined with GRADE score to analyze direct and indirect comparisons, as shown in Fig 3B and Table 2.

**Table 2:** Effect quantity and evidence classification of PSQI.

Comparative measures	Direct comparison		Indirect comparison		Network meta-analysis	
MD(95%CI)	Quality of evidence	MD(95%CI)	Quality of evidence	MD(95%CI)	Quality of evidence
B VS A	−0.14(−3.16, 2.89)	Very Low^**&#**^	−0.83(−3.20,1.55)	Very Low^**&#**^	−0.56 (−2.34,1.23)	Very Low^**&#**^
C VS A	–	–	−2.90 (−6.32,0.52)	Very Low^**&#**^	−2.90 (−6.32,0.52)	Very Low^**&#**^
D VS A	−2.86(−4.27,-1.44)	Very Low^&*^	−3.31(−7.05,0.42)	Very Low^&*^	−2.91 (−4.17,-1.65)	Very Low^&*^
E VS A	−1.79 (−5.22,1.64)	Very Low^**&#**^	Not evaluated^**$**^	Not evaluated^**$**^	−1.79 (−5.22,1.64)	Very Low^**&#**^
F VS A	−3.51(−6.17,-0.85)	Very Low^**&#**^	−0.35(−3.29,2.59)	Very Low^**&#**^	−2.07 (−4.18,0.03)	Very Low^**&#**^
G VS A	−2.23 (−3.87,-0.58)	Very Low^&*^	Not evaluated^**$**^	Not evaluated^**$**^	−2.23 (−3.87,-0.58)	Very Low^&*^
H VS A	−2.15(−4.18,-0.12)	Very Low^**&#**^	−5.90(−12.81,1.02)	Very Low^**&#**^	−2.45 (−4.40,-0.49)	Very Low^**&#**^
I VS A	–	–	−4.36 (−9.82,1.10)	Very Low^**&#**^	−4.36 (−9.82,1.10)	Very Low^**&#**^
J VS A	–	–	−2.61 (−6.35,1.13)	Very Low^**&#**^	−2.61 (−6.35,1.13)	Very Low^**&#**^
C VS B	−2.34 (−5.26,0.58)	Very Low^**&#**^	−1.22(−1239.9,1237.5)	Very Low^**&#**^	−2.34 (−5.26,0.58)	Very Low^**&#**^
D VS B	−2.6(−5.56,0.36)	Very Low^**&#**^	−2.14(−4.83,0.55)	Very Low^**&#**^	−2.35 (−4.24,-0.46)	Very Low^**&#**^
E VS B	–	–	−1.23 (−5.09,2.63)	Very Low^**&#**^	−1.23 (−5.09,2.63)	Very Low^**&#**^
F VS B	−0.52(−2.76,1.71)	Very Low^**&#**^	−3.68(−6.95,0.41)	Very Low^**&#**^	−1.52 (−3.47,0.44)	Very Low^**&#**^
G VS B	–	–	−1.67 (−4.10,0.76)	Very Low^**&#**^	−1.67 (−4.10,0.76)	Very Low^**&#**^
H VS B	−3.29(−6.0,-0.58)	Very Low^**&#**^	0.35(−3.06,3.76)	Very Low^**&#**^	−1.89 (−4.17,0.40)	Very Low^**&#**^
I VS B	−3.8 (−8.96,1.36)	Low^**#**^	−2.68(−1242.13,1236.78)	low^**#**^	−3.80 (−8.96,1.36)	low^**#**^
J VS B	−2.05 (−5.34,1.24)	Very Low^**&#**^	−0.93(−1242.17,1239.31)	Very Low^**&#**^	−2.05 (−5.34,1.24)	Very Low^**&#**^
D VS C	–	–	−0.01 (−3.49,3.46)	Very Low^**&#**^	−0.01 (−3.49,3.46)	Very Low^**&#**^
E VS C	–	–	1.11 (−3.73,5.95)	Very Low^**&#**^	1.11 (−3.73,5.95)	Very Low^**&#**^
F VS C	–	–	0.82 (−2.69,4.34)	Very Low^**&#**^	0.82 (−2.69,4.34)	Very Low^**&#**^
G VS C	–	–	0.67 (−3.12,4.46)	Very Low^**&#**^	0.67 (−3.12,4.46)	Very Low^**&#**^
H VS C	–	–	0.45 (−3.25,4.16)	Very Low^**&#**^	0.45 (−3.25,4.16)	Very Low^**&#**^
I VS C	–	–	−1.46 (−7.39,4.47)	Very Low^**&#**^	−1.46 (−7.39,4.47)	Very Low^**&#**^
J VS C	–	–	0.29 (−4.11,4.69)	Very Low^**&#**^	0.29 (−4.11,4.69)	Very Low^**&#**^
E VS D	–	–	1.12 (−2.53,4.77)	Very Low^**&#**^	1.12 (−2.53,4.77)	Very Low^**&#**^
F VS D	–	–	0.84 (−1.47,3.14)	Very Low^**&#**^	0.84 (−1.47,3.14)	Very Low^**&#**^
G VS D	–	–	0.68 (−1.39,2.75)	Very Low^**&#**^	0.68 (−1.39,2.75)	Very Low^**&#**^
H VS D	–	–	0.47 (−1.78,2.72)	Very Low^**&#**^	0.47 (−1.78,2.72)	Very Low^**&#**^
I VS D	–	–	−1.45 (−6.94,4.05)	Very Low^**&#**^	−1.45 (−6.94,4.05)	Very Low^**&#**^
J VS D	–	–	0.30 (−3.49,4.09)	Very Low^**&#**^	0.30 (−3.49,4.09)	Very Low^**&#**^
F VS E	–	–	−0.28 (−4.31,3.74)	Very Low^**&#**^	−0.28 (−4.31,3.74)	Very Low^**&#**^
G VS E	–	–	−0.44 (−4.24,3.36)	Very Low^**&#**^	−0.44 (−4.24,3.36)	Very Low^**&#**^
H VS E	–	–	−0.66 (−4.60,3.29)	Very Low^**&#**^	−0.66 (−4.60,3.29)	Very Low^**&#**^
I VS E	–	–	−2.57 (−9.02,3.88)	Very Low^**&#**^	−2.57 (−9.02,3.88)	Very Low^**&#**^
J VS E	–	–	−0.82 (−5.89,4.25)	Very Low^**&#**^	−0.82 (−5.89,4.25)	Very Low^**&#**^
G VS F	–	–	−0.15 (−2.83,2.52)	Very Low^**&#**^	−0.15 (−2.83,2.52)	Very Low^**&#**^
H VS F	–	–	−0.37 (−3.05,2.31)	Very Low^**&#**^	−0.37 (−3.05,2.31)	Very Low^**&#**^
I VS F	–	–	−2.28 (−7.80,3.23)	Very Low^**&#**^	−2.28 (−7.80,3.23)	Very Low^**&#**^
J VS F	–	–	−0.53 (−4.36,3.29)	Very Low^**&#**^	−0.53 (−4.36,3.29)	Very Low^**&#**^
H VS G	–	–	−0.22 (−2.77,2.33)	Very Low^**&#**^	−0.22 (−2.77,2.33)	Very Low^**&#**^
I VS G	–	–	−2.13 (−7.83,3.57)	Very Low^**&#**^	−2.13 (−7.83,3.57)	Very Low^**&#**^
J VS G	–	–	−0.38 (−4.47,3.71)	Very Low^**&#**^	−0.38 (−4.47,3.71)	Very Low^**&#**^
I VS H	–	–	−1.91 (−7.56,3.73)	Very Low^**&#**^	−1.91 (−7.56,3.73)	Very Low^**&#**^
J VS H	–	–	−0.16 (−4.17,3.84)	Very Low^**&#**^	−0.16 (−4.17,3.84)	Very Low^**&#**^
J VS I	–	–	1.75 (−4.37,7.87)	Very Low^**&#**^	1.75 (−4.37,7.87)	Very Low^**&#**^

A: CT; B: SS; C: ILF-rTMS; D: LF-rTMS; E: MF-rTMS; F: HF-rTMS; G: L-FES; H: FNS; I: CES; J: tDCS; &: Within-study bias (some concerns); *: Heterogeneity (major concerns);#: Imprecision (major concerns); $: Not evaluated, because all the evidence about these contrasts comes from the trials which directly compare them.

### Network meta-analysis

Ten treatment regimens were subjected to network meta-analysis, and four comparisons showed statistical differences. The decrease in PSQI of LF-rTMS, L-FES, and FNS was higher than that of CT, and the decrease in PSQI of LF-rTMS was higher than that of SS; there was no statistical difference in other comparisons, as shown in Table 3.

**Table 3:** Network meta-analysis of PSQI.

Treatment	tDCS	CES	FNS	L-FES	HF-rTMS	MF-rTMS	LF-rTMS	ILF-rTMS	SS	CT
tDCS	–									
CES	1.75 (−4.37,7.87)	–								
FNS	−0.16 (−4.17,3.84)	−1.91 (−7.56,3.73)	–							
L-FES	−0.38 (−4.47,3.71)	−2.13 (−7.83,3.57)	−0.22 (−2.77,2.33)	–						
HF-rTMS	−0.53 (−4.36,3.29)	−2.28 (−7.80,3.23)	−0.37 (−3.05,2.31)	−0.15 (−2.83,2.52)	–					
MF-rTMS	−0.82 (−5.89,4.25)	−2.57 (−9.02,3.88)	−0.66 (−4.60,3.29)	−0.44 (−4.24,3.36)	−0.28 (−4.31,3.74)	–				
LF-rTMS	0.30 (−3.49,4.09)	−1.45 (−6.94,4.05)	0.47 (−1.78,2.72)	0.68 (−1.39,2.75)	0.84 (−1.47,3.14)	1.12 (−2.53,4.77)	–			
ILF-rTMS	0.29 (−4.11,4.69)	−1.46 (−7.39,4.47)	0.45 (−3.25,4.16)	0.67 (−3.12,4.46)	0.82 (−2.69,4.34)	1.11 (−3.73,5.95)	−0.01 (−3.49,3.46)	–		
SS	−2.05 (−5.34,1.24)	−3.80 (−8.96,1.36)	−1.89 (−4.17,0.40)	−1.67 (−4.10,0.76)	−1.52 (−3.47,0.44)	−1.23 (−5.09,2.63)	−2.35 (−4.24,-0.46)^%^	−2.34 (−5.26,0.58)	–	
CT	−2.61 (−6.35,1.13)	−4.36 (−9.82,1.10)	−2.45 (−4.40,-0.49)^%^	−2.23 (−3.87,-0.58)^%^	−2.07 (−4.18,0.03)	−1.79 (−5.22,1.64)	−2.91 (−4.17,-1.65)^%^	−2.90 (−6.32,0.52)	−0.56 (−2.34,1.23)	–

%There is a statistical difference between the two in comparison

### Publication bias

In this study, 10 treatment regimens were used as outcome indicators of the PSQI. The dots of different colours in the funnel plot represent a direct comparison between two different rehabilitation treatment regimens, and the number of dots represents the number of studies. Linear fitting illustrated that most of the dots in the funnel plot of this study are symmetrically distributed on the vertical line and its two sides, with basic symmetry on both sides. However, a degree of publication bias may still exist. The funnel plot is shown in Fig 3C.

## HAMD

### Evidence network

**Fig 4:**
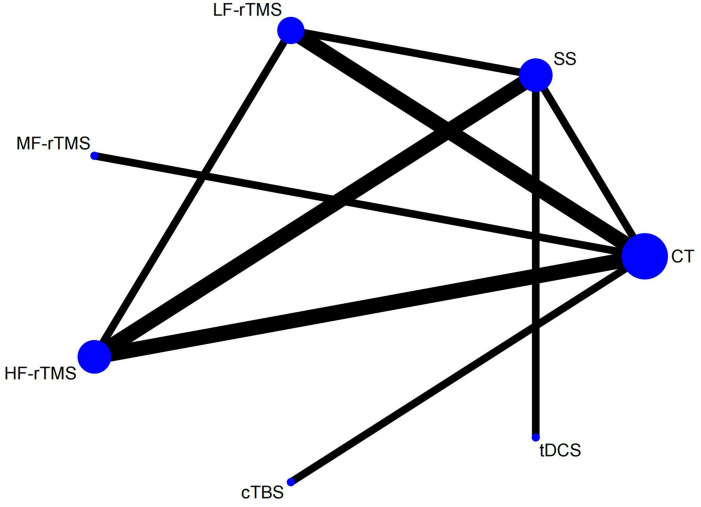
Network diagram of HAMD.

Seven studies involving seven treatment options for HAMD were reported. Three closed loops were formed. The evidence network is shown in Fig 4.

### Inconsistency inspection

There is no simultaneous occurrence of indirect comparison and direct comparison, so there is no need for inconsistency detection, and the consistency model can be used directly.

### Network meta-analysis

Seven treatment plans were subjected to network meta-analysis, and one comparison showed statistical differences. Compared with CT, the decrease in HAMD of MF-rTMS and cTBS was significantly higher than that of CT. Compared with SS, the decrease in HAMD of tDCS, HF-rTMS and LF-rTMS was significantly higher than that of SS. Compared with LF-rTMS, the decrease in HAMD of cTBS and MF-rTMS was significantly lower than that of LF-rTMS. Compared with MF-rTMS, the decrease in HAMD of MF-rTMS and tDCS was significantly higher than that of MF-rTMS. Compared with HF-rTMS, the decrease in HAMD of cTBS was significantly lower than that of HF-rTMS, and there were no statistical differences in the remaining comparisons, as shown in Table 4.

**Table 4:** Network meta-analysis of HAMD.

TREATMENT	tDCS	cTBS	HF-rTMS	MF-rTMS	LF-rTMS	SS	CT
tDCS	–						
cTBS	−3.60 (−7.81,0.61)	–					
HF-rTMS	1.25 (−2.80,5.30)	4.85 (2.65,7.05)^%^	–				
MF-rTMS	−5.36 (−10.20,-0.52)^%^	−1.76 (−4.16,0.64)	−6.61 (−9.86,-3.36)^%^	–			
LF-rTMS	0.75 (−3.28,4.78)	4.35 (2.19,6.51)^%^	−0.50 (−2.33,1.33)	6.11 (2.89,9.33)^%^	–		
SS	−3.90 (−7.02,-0.78)^%^	−0.30 (−3.12,2.52)	−5.15 (−7.73,-2.57)^%^	1.46 (−2.24,5.16)	−4.65 (−7.19,-2.11)^%^	–	
CT	2.47 (−0.68,5.62)	−17.59 (−24.15,-11.03)^%^	0.46 (−1.94,2.86)	−5.65 (−7.80,-3.50)^%^	0.78 (−2.79,4.35)	−1.00 (−3.82,1.82)	–

## NIHSS

### Evidence network

**Fig 5:**
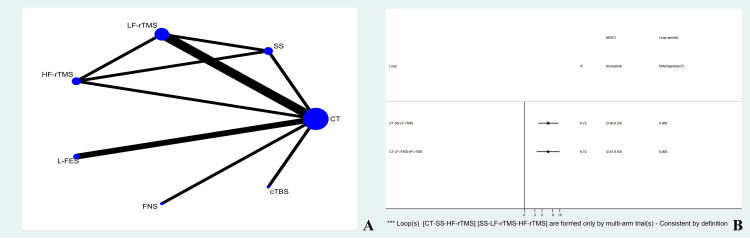
NIHSS ( A: Network diagram of NIHSS; B: NIHSS inconsistency testing).

The NHISS reported seven studies involving seven treatment options. Four closed loops were formed. The number of studies comparing CT with LF-rTMS was the highest (three RCTs). The evidence network is shown in Fig 5A.

### Inconsistency

The seven treatment options for the NHISS formed four triangular loops. The overall inconsistency test results showed P = 0.1211 > 0.05, indicating no overall inconsistency. The inconsistent factors were 6.72 (CT-SS-LF-rTMS) with 95% CI of (3.90, 9.53) and 6.72 (CT-LFrTMS-HF-rTMS) with 95% CI of (3.51, 9.93). In this study, there were two closed-loops, and 95% CI values did not reach zero. We used the node splitting method combined with GRADE score to analyze direct and indirect comparisons, as shown in Fig 5B and Table 5.

**Table 5:** Effect quantity and evidence classification of NHISS.

Comparative measures	Direct comparison		Indirect comparison		Network meta-analysis	
MD(95%CI)	Quality of evidence	MD(95%CI)	Quality of evidence	MD(95%CI)	Quality of evidence
B VS A	−1.05(−7.34, 5.24)	Very Low^**&#**^	−13.27(−27.36,0.82)	Very Low^**&#**^	−3.08 (−10.05,3.89)	Very Low^**&#**^
C VS A	–	–	−5.11 (−9.53,-0.68)	Very Low^&*@^	−5.11 (−9.53,-0.68)	Very Low^&*@^
D VS A	−4.5(−10.88,1.88)	Very Low^**&#**^	−16.72(−30.85,-2.59)	Very Low^**&#**^	−6.53 (−13.59,0.52)	Very Low^**&#**^
E VS A	–	–	−2.65 (−7.98,2.68)	Very Low^**&#@**^	−2.65 (−7.98,2.68)	Very Low^**&#@**^
F VS A	–	–	−1.08 (−8.66,6.50)	Very Low^**&#@**^	−1.08 (−8.66,6.50)	Very Low^**&#@**^
G VS A	–	–	0.97 (−6.46,8.40)	Very Low^**&#@**^	0.97 (−6.46,8.40)	Very Low^**&#@**^
C VS B	1.19E-09(−6.28,6.28)	Very Low^**&#**^	−12.22(−26.32,1.88)	Very Low^**&#**^	−2.02 (−8.99,4.94)	Very Low^**&#**^
D VS B	–	–	−3.45 (−11.15,4.25)	Very Low^**&#@**^	−3.45 (−11.15,4.25)	Very Low^**&#@**^
E VS B	–	–	0.43 (−8.35,9.21)	Very Low^**&#@**^	0.43 (−8.35,9.21)	Very Low^**&#@**^
F VS B	–	–	2.00 (−8.30,12.30)	Very Low^**&#@**^	2.00 (−8.30,12.30)	Very Low^**&#@**^
G VS B	–	–	4.05 (−6.14,14.24)	Very Low^**&#@**^	4.05 (−6.14,14.24)	Very Low^**&#@**^
D VS C	−3.45(−9.82,2.92)	Very Low^**&#**^	8.77(−5.37,22.91)	Very Low^**&#**^	−1.43 (−8.48,5.63)	Very Low^**&#**^
E VS C	–	–	2.46 (−4.47,9.38)	Very Low^**&#@**^	2.46 (−4.47,9.38)	Very Low^**&#@**^
F VS C	–	–	4.03 (−4.75,12.80)	Very Low^**&#@**^	4.03 (−4.75,12.80)	Very Low^**&#@**^
G VS C	–	–	6.08 (−2.57,14.73)	Very Low^**&#@**^	6.08 (−2.57,14.73)	Very Low^**&#@**^
E VS D	–	–	3.88 (−4.96,12.72)	Very Low^**&#@**^	3.88 (−4.96,12.72)	Very Low^**&#@**^
F VS D	–	–	5.45 (−4.90,15.81)	Very Low^**&#@**^	5.45 (−4.90,15.81)	Very Low^**&#@**^
G VS D	–	–	7.50 (−2.75,17.75)	Very Low^**&#@**^	7.50 (−2.75,17.75)	Very Low^**&#@**^
F VS E	–	–	1.57 (−7.70,10.84)	Very Low^**&#@**^	1.57 (−7.70,10.84)	Very Low^**&#@**^
G VS E	–	–	3.62 (−5.53,12.77)	Very Low^**&#@**^	3.62 (−5.53,12.77)	Very Low^**&#@**^
G VS F	–	–	2.05 (−8.57,12.67)	Very Low^**&#@**^	2.05 (−8.57,12.67)	Very Low^**&#@**^

A: CT; B: SS; C: LF-rTMS; D: HF-rTMS; E: L-FES; F: FNS; G: cTBS; &: Within-study bias (Some concerns); *: Heterogeneity (Major concerns);#: Imprecision (Major concerns);@: Incoherence (Some concerns)

### Network meta-analysis

Seven treatment plans were subjected to network meta-analysis, and one comparison showed statistically significant differences. Compared with CT, the degree of NHISS decrease in the LF-rTMS group was higher, and there was no statistical difference in the other comparisons, as shown in Table 6.

**Table 6:** Network meta-analysis of NHISS.

TREATMENT	cTBS	FNS	L-FES	HF-rTMS	LF-rTMS	SS	CT
cTBS	–						
FNS	2.05 (−8.57,12.67)	–					
L-FES	3.62 (−5.53,12.77)	1.57 (−7.70,10.84)	–				
HF-rTMS	7.50 (−2.75,17.75)	5.45 (−4.90,15.81)	3.88 (−4.96,12.72)	–			
LF-rTMS	6.08 (−2.57,14.73)	4.03 (−4.75,12.80)	2.46 (−4.47,9.38)	−1.43 (−8.48,5.63)	–		
SS	4.05 (−6.14,14.24)	2.00 (−8.30,12.30)	0.43 (−8.35,9.21)	−3.45 (−11.15,4.25)	−2.02 (−8.99,4.94)	–	
CT	0.97 (−6.46,8.40)	−1.08 (−8.66,6.50)	−2.65 (−7.98,2.68)	−6.53 (−13.59,0.52)	−5.11 (−9.53,-0.68)^%^	−3.08 (−10.05,3.89)	–

### Clinical total effective rate

#### Evidence network and inconsistency testing.

**Fig 6:**
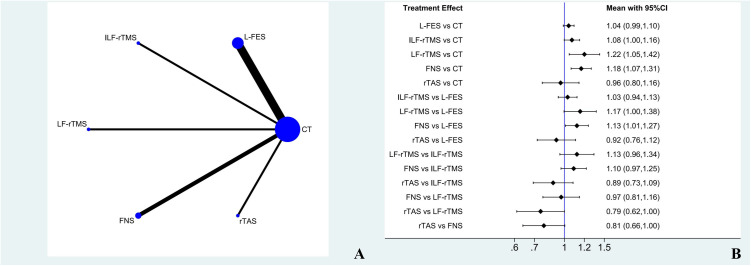
Clinical total effective rate ( A: Network diagram of Clinical total effective rate; B: Forest plot of Clinical total effective rate).

Nine studies reported the total clinical response rates involving seven treatment regimens. Inconsistency testing was not required because a closed loop was not formed. The evidence network for total clinical effectiveness is shown in Fig 6A.

### Network meta-analysis

Seven treatment plans were subjected to network meta-analysis, and three comparisons showed statistical differences. Compared with CT, the clinical total effective rate of LF-rTMS and FNS was significantly higher. Compared with L-FES, the clinical total effective rate of FNS was superior, and there was no statistical difference in other comparisons, as shown in Fig 6B.

## SUCRA

### PSQI

**Fig 7:**
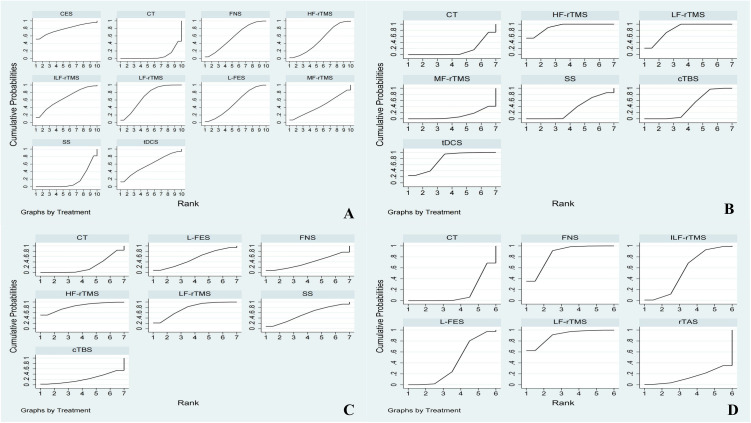
SUCRA ( A: PSQI; B: HAMD; C: NHISS; D: Clinical total effective rate).

SUCRA probability ranking results were as follows: CES (78.2%)>LF-rTMS (69.6%)>ILF-rTMS (64.6%)>tDCS (59%)>FNS (58.1%)>L-FES (52.6%)>HF-rTMS (49.9%)>MF-rTMS (44.3%)>SS (16.3%)>CT (7.4%). The cumulative probability ranking diagram is shown in Fig 7A; the larger the area under the curve, the more effective it is.

### HAMD

The SUCRA probability ranking results were as follows: HF-rTMS (90.5%)>LF-rTMS (82.3%)>tDCS (75.7%)>cTBS (42.8%)>SS (32.6%)>CT (15.0%)>MF-rTMS (11.0%). The cumulative probability-ranking diagram is shown in Fig 7B.

### NHISS

The SUCRA probability ranking results were as follows: HF-rTMS (83.3%)>LF-rTMS (75.6%)>SS (54.4%)>FES (52.5%)>EBS (38.2%)>CT (23.5%)>cTBS (22.5%). A cumulative probability ranking diagram is shown in Fig 7C.

### Clinical total effective rate

The SUCRA probability ranking results were as follows: LF-rTMS (90%)>FNS (85%)>ILF-rTMS (54.8%)>L-FES (40.6%)>CT (15%)>rTAS (14.6%). The cumulative probability-ranking diagram is shown in Fig 7D.

### Safety

Among the included studies, four reported adverse reactions [18, 23, 27, 37]. The intervention methods used to treat the various degrees of adverse reactions were CT, SS, LF-rTMS, and ILF-rTMS. The main manifestations of adverse reactions include dizziness, headache, drowsiness, fatigue, shoulder hand syndrome, ataxia, infection, upper-limb spasms, nausea, palpitations, and shortness of breath. The specific adverse reactions are shown in Table 7. The number of adverse reactions in the CT group was 33, with nine types of adverse reactions (dizziness, headache, drowsiness, dizziness, fatigue, shoulder hand syndrome, ataxia, infection, upper limb spasms). Among them, the number of adverse reactions in the SS group was two, with three types of adverse reactions (nausea, palpitations, shortness of breath). The number of adverse reactions in the LF-rTMS group was nine, with four types of adverse reactions (dizziness, headache, dizziness, and fatigue). The number of adverse reactions in the ILF-rTMS group was eight, with two types of adverse reactions (ataxia and upper limb spasms).

**Table 7:** Analysis of adverse reactions (number of events).

Treatment	dizziness	headache	drowsiness	vertigo	fatigue	shoulder hand syndrome	ataxia	infection	upper limb spasms	nausea	palpitations and shortness of breath
CT	4	4	9	2	3	2	3	2	4	–	–
LF-rTMS	2	3	0	3	1	–	–	–	–	–	–
SS	–	–	–	0	0	–	–	–	–	1	1
ILF-rTMS	–	–	–	–	–	0	4	0	4	0	0

## Discussion

Quality sleep significantly impacts recovery in the sequelae of many cardiovascular and cerebrovascular diseases, highlighting its relevance in PSI treatment. Currently, conventional anti-insomnia drugs can only alleviate the clinical symptoms of some PSI patients, while electromagnetic stimulation therapy has gradually become an emerging complementary and alternative therapy for PSI owing to its good efficacy and safety. The etiology of insomnia after stroke is very complex. The etiology is mainly divided into physiological and psychological differences. Studies have shown that changes in a variety of neurotransmitters related to sleep (such as 5-HT and norepinephrine neurotransmitters) in the body of stroke patients during the onset can eventually cause insomnia [41]. Secondly, anatomically, if the injury of stroke patients involves the ascending reticular system of the brain that is related to awakening, the patients will also suffer from insomnia due to the reduction of awakening ability [42]. Relevant studies have also shown that [42–44] severe lethargy may be related to the involvement of the thalamus, midbrain, pons and changes in cerebral blood flow, blood flow velocity and blood volume at the stroke site, and the specific mechanism still needs further study. In terms of psychological factors, after the onset of stroke, the daily self-care ability of stroke patients is seriously affected, which leads to the generation of negative emotions such as anxiety and depression, and further exacerbates insomnia symptoms. Even some long-term bedridden patients may suffer from insomnia due to the reversal of day and night sleep, that is, excessive sleep during the day and insufficient sleep at night [45]. And stroke patients in the acute onset period may also experience pain due to changes in muscle tone or central nervous system abnormalities after stroke, further affecting sleep quality. At present, Modern medical treatment for insomnia after stroke often uses sedative and hypnotic drugs or antidepressants and anxiolytics. Most of these drugs are accompanied by many side effects, such as respiratory arrest, drug dependence and tolerance, exacerbation of motor and cognitive dysfunction, etc. [46], while electrical stimulation therapy, as a supplementary or alternative therapy with fewer side effects and better efficacy, can increase efficacy while replacing a certain degree of drug use, avoiding drug resistance and side effects caused by excessive use. This study involved 10 electromagnetic therapies, including L-FES, ILF-rTMS, LF-rTMS, FNS, rTAS, MF-rTMS, HF-rTMS, CES, tDCS, and cTBS, excluding the CT and SS groups. Of which, ILF-rTMS, LF-rTMS, MF-rTMS, and HF-rTMS represent different frequency modes of rTMS treatment. cTBS is another stimulation mode of TMS; and both tDCS and TMS are noninvasive neuromodulatory techniques. tDCS modulates the neuronal membrane potential through constant, low-intensity direct current to alter cortical excitability [47]. rTAS is a revolutionary and innovative acupuncture treatment, with the same starting point as that of rTMS technology. By applying stimulation outside the skull to generate a specific field strength, it directly affects corresponding brain regions, thereby enhancing brain plasticity and triggering changes in synapses, ion channels, membrane potentials. L-FES mimics neural impulses by controlling low-frequency electrical stimulation and alters resting membrane potential by stimulating the cerebral cortex. FNS operates on the principle of applying low-frequency pulse currents of a specific intensity to stimulate the brain following a predetermined program, thereby improving or restoring tissue function [48]. CES stimulates the central nervous system through microcurrent stimulation, improves abnormal brain waves, induces the enhancement of brain waves that stabilize emotions, and regulates various neurotransmitters and hormones in the brain, thereby alleviating insomnia symptoms [49]. This study combined the results of previous RCTs with network meta-analysis methods to evaluate the clinical efficacy and safety of different electromagnetic therapies for PSI and ranked them based on their efficacy. There has also been some meta-analysis of the clinical efficacy of brain stimulation in the treatment of post-stroke sleep in the past. Huang [50] used traditional meta-analysis methods to study RCTs of previous noninvasive brain stimulation in the treatment of post-stroke sleep. The results showed that noninvasive brain stimulation could effectively improve the sleep quality, structure, depression level and BDNF level of PSI patients, and it was also safe. However, this study is based on the traditional meta-analysis, using the network meta-analysis method to make pairwise comparisons between different electromagnetic stimulation therapies, and using SUCRA to sort, which has certain clinical guiding significance; At the same time, different from Huang’s study, this study uses the difference between before and after treatment to analyze, which can avoid the problem of different baseline before treatment to a certain extent.

The PSQI was developed in 1989 by Dr. Buysse, a psychiatrist at the University of Pittsburgh, and his colleagues in the United States [51]. This scale is suitable for evaluating sleep quality in both patients with sleep and mental disorders and the general population. It is often used to assess the sleep quality of participants over the past month. It consists of 19 self-evaluations and five additional evaluation items, with a total score range of 0–21. Higher scores indicate poorer sleep quality. Many clinical trials related to sleep disorders have used the PSQI as one of the main indicators. The aetiology of PSI is often related not only to lesions in the brain parenchyma but also to the degree of post-stroke depression. Another key indicator used in this study, the HAMD, was developed by Hamilton in 1960 and is the most used scale for the clinical assessment of depression [52]. The combined evaluation of PSQI and HAMD provides a more comprehensive understanding of the sleep and emotional status of PSI patients. In this study, the SUCRA ranking results of PSQI were as follows: CES, LF-rTMS, ILF-rTMS, HF-rTMS, LF-rTMS, tDCS for HAMD and LF-rTMS, FNS, ILF-rTMS for clinical total efficacy. Based on the ranking results of three outcome indicators, LF-rTMS was significantly superior to other electromagnetic therapies in relieving insomnia in stroke patients. The basic principle of rTMS is based on Faraday’s electromagnetic induction principle: a time-varying current generates a time-varying magnetic field when it passes through a coil vertically placed on the head, which causes neurones to sense the induced current and produce a series of biological effects to achieve the treatment goal. RTMS primarily uses different frequencies to achieve the treatment goals. To improve brain function in patients with various diseases, an optimal treatment effect can be achieved by adjusting the stimulation intensity, frequency, location, and coil direction [53–54]. In this study, rTMS was divided into four frequency modes based on frequency, namely ILF-rTMS (0.01 Hz), LF-rTMS (1 Hz), MF-rTMS (5 Hz), HF-rTMS (10 Hz). High frequency of TMS produces excitatory effects, while low frequency produces inhibitory effects; rTMS modifies neural function at multiple sites through connections and interactions, influencing local nerves. In this study, among all rTMS frequency patterns that improved the sleep quality of PSI patients, LF-rTMS had the most outstanding therapeutic effect. Controversy surrounds the efficacy of HF-rTMS and LF-rTMS in PSI treatment [53], and ongoing research is exploring their mechanisms of action. However, this study’s results suggest potential PSI treatment options. Inhibitory rTMS has better therapeutic effects compared to the promoting rTMS phase. Nonetheless, the connections that affect the cerebral cortex and related brain regions still require further large-scale, high-quality RCTs and basic research to unravel their mechanisms of action.

NIHSS is the neurological function assessment scale designed by Thomos et al. in 1989 for the treatment of acute stroke. It consists of selecting meaningful items from three scales (Toronto Stroke Scale, Oxford Initial Severity Scale, Cincinnati Stroke Scale) [55]. Currently, it is the most used scoring system in clinical neuropathy scoring, which describes the severity of nerve injury through scores. There are seven intervention methods that involved NIHSS in this study, namely HF-rTMS, LF-rTMS, SS, FES, EBS, CT, and cTBS. The ranking results of the NIHSS and SUCRA showed that HF-rTMS was the most effective intervention method. Some researchers have conducted prospective observations on the efficacy of HF-rTMS in treating acute spontaneous cerebral haemorrhage, and the results revealed absence of epilepsy cases or NIHSS deterioration in patients after rTMS intervention. HF-rTMS was independently associated with a good outcome at 3 months, and its mechanism may be related to the enhancement of focal neural activity in the cerebral cortex by HF-rTMS [56].

### Safety

There were few reports (n = 4) related to adverse reactions in this study. Most other studies either did not mention adverse reactions or mentioned that there were no adverse reactions in any group. Although electromagnetic stimulation therapy often has high safety at appropriate stimulation frequencies, there are numerous low-quality studies included in the RCTs that do not use adverse reactions as outcome indicators. The results of this study showed that, in addition to CT and SS therapy, LF-rTMS had the most types of adverse reactions, including dizziness, headache, and fatigue, whereas ILF-rTMS had fewer types of adverse reactions, including ataxia and upper limb spasms. Owing to the limited literature and the absence of exclusion of differences in the course of treatment or other factors, the adverse reaction results should be considered for reference only.

## Limitations

To the best of our knowledge, this is the first study to conduct network meta-analysis on PSI treatment with different electromagnetic therapies. Simultaneously, we refined the classification based on different frequency patterns of the same therapy, and the results can serve as a reference for clinical workers. This study had some limitations: 1. The CT group was not refined, and most of the included studies only mentioned routine treatment and did not provide detailed information on the drugs used in routine treatment. Different drug interventions have different therapeutic effects, which may lead to biased results. 2. The quality of the studies included in this study was relatively low, and many literature did not mention randomization, blinding, or allocation concealment methods, which may lead to significant bias in the results. 3. This article did not conduct subgroup analysis, mainly because the number of studies included was not very high, and the quality of most studies was low. Basic information such as stroke site and onset time were not detailed, which may increase the possibility of inconsistency and clinical heterogeneity. After all, detailed subgroup analysis based on the stage or location of stroke can provide more meaningful clinical guidance, and we cannot deny that this is one of the biggest issues in our meta-analysis. 4.Some studies did not mention adverse reactions, which is not conducive to the safety evaluation of drugs. Therefore, the quality of original research should be valued, and RCT reports should strictly adhere to the unified standards for experimental reports, which can improve the authenticity of network meta-analysis. 5. It is worth noting that some of these studies have also compared the different efficacy of drug combined with electrical stimulation therapy with that of the drug alone group or the drug free group, which will also lead to the deviation of the results to some extent, because it is difficult for us to distinguish from the results whether its effectiveness is the main effect of electrical stimulation or the synergistic effect with drugs. Therefore, further clearly controlling variables between groups can more clearly distinguish the sources of their effectiveness.

## Conclusions

Electromagnetic stimulation therapy improved the sleep quality of PSI patients to varying degrees, with rTMS showing the most significant clinical efficacy. Among rTMS, LF-rTMS demonstrated the best clinical efficacy in PSI. Compared to other therapies, HF-rTMS can better improve the neurological damage caused by stroke. However, although this study shows that LF-rTMS and HF-rTMS have different degrees of advantages in the treatment of PSI, it is worth mentioning that because the quality of evidence of RCTs included in this study is generally low, and the sample is small, it is easy to cause the risk of bias, so that it is insufficient for us to make clear clinical recommendations, and the efficacy efficacy and the statistics of adverse reactions could not fully explain the advantages and disadvantages of clinical efficacy and safety and can only be used as clinical references.In the future, more center, large sample, double-blind, clinical randomized controlled trials are needed to verify the results of this study.

## Supporting information

S1 File List of retrieved studies. (XLSX)

S2 File Cochrance quality assessment scale. (XLSX)

S3 File Data. (XLSX)

S4 File Appendix. (DOC)

S5 File PRISMA 2020 checklist. (DOCX)
