# Supplementary material for: Effectiveness and safety of different electromagnetic stimulation therapies in treating post-stroke insomnia: A network meta-analysis of randomized controlled trials
Source: PLoS One. 2025 Jul 3;20(7):e0327544. doi: 10.1371/journal.pone.0327544 (PMC12225873; doi:10.1371/journal.pone.0327544)
Supplement: S4 File — (DOC) [file pone.0327544.s004.doc]

**DATE：**1900/01/01-2024/09/01

**PUBMED:**

1#(Disorders of Initiating[Title/Abstract] AND Maintaining Sleep[Title/Abstract]) OR (DIMS (Disorders of Initiating[Title/Abstract] AND Maintaining Sleep[Title/Abstract]))) OR (Early Awakening[Title/Abstract])) OR (Awakening, Early[Title/Abstract])) OR (Insomnia[Title/Abstract])) OR (Sleep Initiation Dysfunction[Title/Abstract])) OR (Dysfunction, Sleep Initiation[Title/Abstract])) OR (Dysfunctions, Sleep Initiation[Title/Abstract])) OR (Sleep Initiation Dysfunctions[Title/Abstract])) OR (Sleeplessness[Title/Abstract])) OR (Insomnias[Title/Abstract])

2# (clinical[tiab] AND trial[tiab]) OR "clinical trials as topic"[mesh] OR "clinical trial"[pt] OR random*[tiab] OR "random allocation"[mesh] OR "therapeutic use"[sh]

3#Stroke[Title/Abstract] OR Strokes[Title/Abstract] OR Cerebrovascular Accident[Title/Abstract] OR Cerebrovascular Accidents[Title/Abstract] OR Cerebrovascular Apoplexy[Title/Abstract] OR Apoplexy, Cerebrovascular[Title/Abstract] OR Vascular Accident, Brain[Title/Abstract] OR Cerebrovascular Stroke[Title/Abstract] OR Cerebrovascular Strokes[Title/Abstract] OR Stroke, Cerebrovascular[Title/Abstract]

1# AND 2# AND 3#

197

**EMBASE:**

1#('strokes':ab,ti OR 'stroke':ab,ti OR 'cerebrovascular accidents':ab,ti OR 'cerebrovascular apoplexy':ab,ti OR 'apoplexy, cerebrovascular':ab,ti OR 'vascular accident, brain':ab,ti OR 'cerebrovascular stroke':ab,ti OR 'cerebrovascular strokes':ab,ti OR 'stroke, cerebrovascular':ab,ti OR 'cerebrovascular accident':ab,ti)

2#('disorders of initiating and maintaining sleep':ab,ti OR 'dims (disorders of initiating and maintaining sleep)':ab,ti OR 'early awakening':ab,ti OR 'awakening, early':ab,ti OR 'insomnia':ab,ti OR 'sleep initiation dysfunction':ab,ti OR 'dysfunction, sleep initiation':ab,ti OR 'sleep initiation dysfunctions':ab,ti OR 'sleeplessness':ab,ti OR 'insomnias':ab,ti)

3#'randomized controlled trial'/exp OR 'controlled clinical trial'/exp OR randomized:ti,ab OR placebo:ti,ab OR 'drug therapy':lnk OR randomly:ti,ab OR trial:ti,ab OR groups:ti,ab

4#[<1966-2024]/py

1# AND 2# AND 3#

338

**Cochrane Library Central Register of Controlled Trials**

#1(Strokes):ti,ab,kw OR (stroke):ti,ab,kw OR (Cerebrovascular Accident):ti,ab,kw OR (Cerebrovascular Accidents):ti,ab,kw OR (Cerebrovascular Apoplexy):ti,ab,kw OR (Apoplexy, Cerebrovascular):ti,ab,kw OR (Vascular Accident, Brain):ti,ab,kw OR (Cerebrovascular Stroke):ti,ab,kw OR (Cerebrovascular Strokes):ti,ab,kw OR (Stroke, Cerebrovascular):ti,ab,kw

#2 (Disorders of Initiating and Maintaining Sleep):ti,ab,kw OR (DIMS (Disorders of Initiating and Maintaining Sleep)):ti,ab,kw OR (Early Awakening):ti,ab,kw OR (Awakening, Early):ti,ab,kw OR (Insomnia):ti,ab,kw OR (Sleep Initiation Dysfunction):ti,ab,kw OR (Dysfunction, Sleep Initiation):ti,ab,kw OR (Sleep Initiation Dysfunctions):ti,ab,kw OR (Sleeplessness):ti,ab,kw OR (Insomnias):ti,ab,kw

#1 AND #2

313

**APA PsycInfo**

S1:AB strokes OR AB stroke OR AB cerebrovascular accidents OR AB cerebrovascular apoplexy OR AB apoplexy, cerebrovascular OR AB vascular accident, brain OR AB cerebrovascular stroke OR AB cerebrovascular strokes OR AB stroke, cerebrovascular OR AB cerebrovascular accident

S2:AB ( Disorders of Initiating and Maintaining Sleep ) OR AB ( DIMS (Disorders of Initiating and Maintaining Sleep) ) OR AB Early Awakening OR AB Awakening, Early OR AB Insomnia OR AB Sleep Initiation Dysfunction OR AB Dysfunction, Sleep Initiation OR AB Sleep Initiation Dysfunctions OR AB Sleeplessness OR AB Insomnias

S1 AND S2

189

**SinoMed：**

("脑梗死"[摘要:智能] OR "脑出血"[摘要:智能] OR "卒中"[摘要:智能] OR "中风"[摘要:智能] OR "脑缺血"[摘要:智能]) AND ("睡眠"[摘要:智能] OR "失眠"[摘要:智能]) AND "随机"[摘要:智能]

2278

**China national knowledge infrastructure database (CNKI):**

TKA=('卒中'+'脑缺血'+'脑梗死'+'脑出血'+'中风') AND TKA=('睡眠'+'失眠') AND TKA='随机'

1437

**WANFANG:**

主题:(卒中 or 脑缺血 or 脑梗死 or 脑出血 or 中风) and 主题:(睡眠 or 失眠) and 主题:(随机)

2557
